# Complex Rearrangement of the Entire Retinal Posterior Pole in Patients with Relapsing Remitting Multiple Sclerosis

**DOI:** 10.3390/jcm10204693

**Published:** 2021-10-13

**Authors:** Alessio Martucci, Doriana Landi, Massimo Cesareo, Emiliano Di Carlo, Giovanni Di Mauro, Roberto Pietro Sorge, Maria Albanese, Carolina Gabri Nicoletti, Giorgia Mataluni, Nicola Biagio Mercuri, Matteo Di Marino, Francesco Aiello, Diego Centonze, Carlo Nucci, Girolama Alessandra Marfia, Raffaele Mancino

**Affiliations:** 1 Ophthalmology Unit, Department of Experimental Medicine, University of Rome Tor Vergata, Via Montpellier, 1, 00133 Rome, Italy; alessio.martucci@live.it (A.M.); massimo.cesareo@uniroma2.it (M.C.); emi.dicarlo@hotmail.it (E.D.C.); matteo_di_marino@msn.com (M.D.M.); francescoaiello@hotmail.com (F.A.); nucci@med.uniroma2.it (C.N.); mancino@med.uniroma2.it (R.M.); 2 Multiple Sclerosis Clinical and Research Unit, Fondazione PTV Policlinico Tor Vergata, Viale Oxford, 81, 00133 Rome, Italy; doriana.landi@gmail.com (D.L.); giovannidimauro92@gmail.com (G.D.M.); carolgabri@gmail.com (C.G.N.); giorgia.mataluni@gmail.com (G.M.); centonze@uniroma2.it (D.C.); 3 Department of Systems Medicine, University of Rome Tor Vergata, Via Montpellier, 1, 00133 Rome, Italy; mercuri@med.uniroma2.it; 4 Städtisches Klinikum Karlsruhe, Moltkestraße 90, 76133 Karlsruhe, Germany; 5 Neurology Unit, Fondazione PTV Policlinico Tor Vergata, Viale Oxford, 81, 00133 Rome, Italy; maria.albanese@hotmail.it; 6 Laboratory of Biometry, Department of Systems Medicine, University of Rome Tor Vergata, Via Cracovia, 50, 00133 Rome, Italy; sorge@uniroma2.it; 7 Unit of Neurology, IRCCS Istituto Neurologico Mediterraneo NEUROMED, Via Atinense, 18, 86077 Pozzilli, Italy; 8 Multiple Sclerosis Clinical and Research Unit, Tor Vergata Hospital, Viale Oxford, 81, 00133 Rome, Italy

**Keywords:** retina, multiple sclerosis, OCT, posterior pole analysis, optic nerve (ON)

## Abstract

There are consolidated data about multiple sclerosis (MS)–dependent retinal neurodegeneration occurring in the optic disk and the macula, although it is unclear whether other retinal regions are affected. Our objective is to evaluate, for the first time, the involvement of the entire retinal posterior pole in patients diagnosed with relapsing remitting multiple sclerosis (RRMS) unaffected by optic neuritis using Spectral Domain–Optical Coherence Tomography (SD–OCT). The study protocol was approved by Tor Vergata Hospital Institutional Ethics Committee (Approval number 107/16), and conforms to the tenets of the Declaration of Helsinki. After a comprehensive neurological and ophthalmological examination, 53 untreated RRMS patients (aged 37.4 ± 10) and 53 matched controls (aged 36.11 ± 12.94) were enrolled. In addition, each patient underwent an examination of the posterior pole using the SD-OCT built-in Spectralis posterior pole scanning protocol. After segmentation, the mean thickness, as well as the thickness of the 64 single regions of interest, were calculated for each retinal layer. No statistically significant difference in terms of average retinal thickness was found between the groups. However, MS patients showed both a significantly thinner ganglion cell layer (*p* < 0.001), and, although not statistically significant, a thinner inner nuclear layer (*p* = 0.072) and retinal nerve fiber layer (*p* = 0.074). In contrast, the retinal pigment epithelium (*p* = 0.014) and photoreceptor layers *p* < 0.001) resulted significantly thicker in these patients. Interestingly, the analysis of the region of interest showed that neurodegeneration was non-homogeneously distributed across each layer. This is the first report that suggests a complex rearrangement that affects, layer by layer, the entire retinal posterior pole of RRMS retinas in response to the underlying neurotoxic insult.

## 1. Introduction

Multiple sclerosis (MS) is a chronic inflammatory disease of the central nervous system, caused by a profound dysregulation of the adaptive immune system. The immune-mediated damage causes demyelination and neurodegeneration, which are considered to be pathological hallmarks of the disease. These two processes, although mutually linked, also occur independently as a result of different pathogenetic mechanisms that have not yet been fully established. Inflammatory demyelination and neurodegeneration frequently affect the visual system, including the optic nerve and the retina. Several studies investigating retinal damage in MS by using optical coherence tomography (OCT) have consistently found a thinning of the peripapillary retinal nerve fiber layer (pRNFL), the macular ganglion cell layer (GCL), and the inner plexiform layer (IPL) [1]. These effects are more pronounced in eyes affected by optic neuritis (ON), yet are also visible in non-ON eyes. Nevertheless, these studies almost exclusively focused on two retinal regions, the optic disk and the macula, mostly due to technical limitations of older OCT scanners. Therefore, it is still unknown whether retinal regions outside the optic disk and the macula may be also affected by neurodegenerative processes, and to what extent.

Spectral domain (SD)-OCT allows for measuring the entire retinal posterior pole thickness at each retinal layer. This instrument has been proved to be sensitive enough to detect neurodegeneration in other ocular diseases, such as autosomal dominant optic atrophy (ADOA), glaucoma, Parkinson’s disease, and MS [2,3,4,5,6].

This study aims to investigate the retinal posterior pole in untreated patients affected by relapsing remitting MS (RRMS), without clinical or electrophysiological evidence of ON, compared to a control group of healthy individuals, by using Spectralis SD-OCT.

## 2. Materials and Methods

In this case-control monocentric study, 53 patients affected by RRMS and 53 healthy subjects (controls) were included. Patients were enrolled from the Multiple Sclerosis Center of the Tor Vergata University Hospital, Rome, Italy. Healthy control individuals were enrolled from the outpatient clinic of the Ophthalmology Unit of the Tor Vergata University Hospital, Rome, Italy.

Inclusion criteria for the study were: diagnosis of RRMS, according to the revised McDonald criteria (2010) [7]; best corrected visual acuity 0,0 logMar; and not being under MS disease modifying treatments. Exclusion criteria were: retinal and optic nerve diseases; previous ocular surgical procedures; use of any toxic drug therapy for the retina and/or the optic nerve; spherical refractive defects greater than 3 diopters and cylindrical refractive defects greater than 2 diopters; and the presence of severe nystagmus. Eyes currently or previously affected by optic neuritis were also excluded. The following parameters were collected for each subject included in the study: age, gender, disease duration (interval between onset of MS and OCT examination, measured in months), and Expanded Disability Status Scale (EDSS) at the time of acquisition of OCT.

The study protocol was approved by Tor Vergata Hospital Institutional Ethics Committee (approval number 107/16) and conforms to the tenets of the Declaration of Helsinki. All subjects recruited in this study provided written informed consent to the procedures.

All participants underwent a comprehensive ophthalmological examination and OCT scan (SD-OCT, Spectralis, Heidelberg Engineering, Heidelberg, Germany) of the retinal posterior pole and the pRNFL. Data were collected from both eyes of each participant.

**Figure 1:**
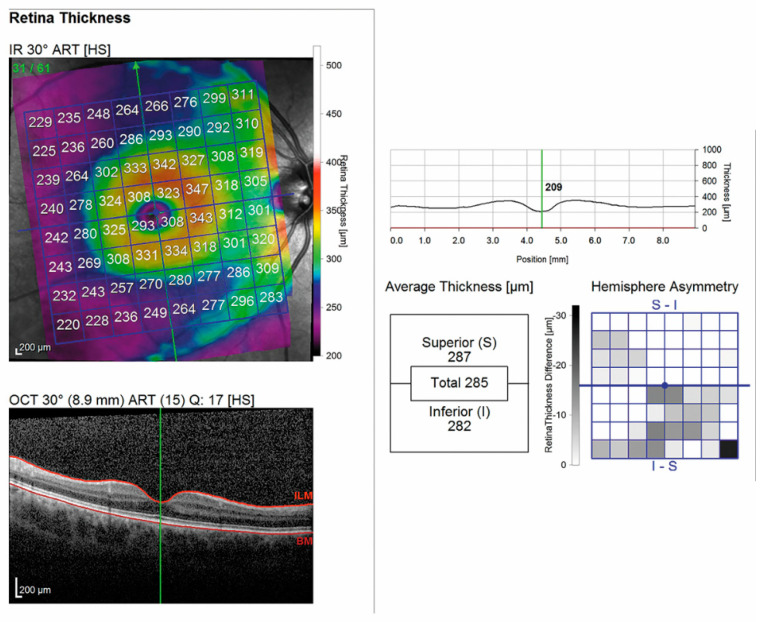
OCT scan of the posterior pole in a patient affected by MS.

All SD-OCT scans were acquired by the same experienced operator after pupil dilation with 0.5% tropicamide and 10% phenylephrine eye drops (Visumidriatic Fenilefrina, Visufarma). Posterior pole measurements from each SD-OCT scan were performed using the built-in Spectralis software Heidelberg Eye Explorer (version 6.0c, Heidelberg Engineering, Heidelberg, Germany), as previously described [2]. The Spectralis vertical posterior pole scanning protocol (scanning area: 30° × 25°), comprising 61 single vertical scans centered on the fovea, was used to obtain volumetric retinal scans. The retinal thickness grid overlayed a 24° × 24° retinal region centered on the measured area. This grid was composed of 64 volumetrics units; each VU represented the average measured retinal thickness of a 3° × 3° area (Figure 1).

The Spectralis segmentation software was used to obtain the following thickness measurements of the vertical posterior pole (pp) scanning protocol: total retinal thickness (RETINA); retinal nerve fiber layer (ppRNFL); ganglion cell layer (ppGCL); inner plexiform layer (ppIPL); inner nuclear layer (ppINL); outer plexiform layer (ppOPL); outer nuclear layer (ppONL); retinal pigment epithelium (ppRPE); and photoreceptors (ppPHOTO). Posterior pole thickness values of the inner retinal layers (ppIRL) and outer retinal layers (ppORL) were also collected through the automatic segmentation tool.

**Figure 2:**
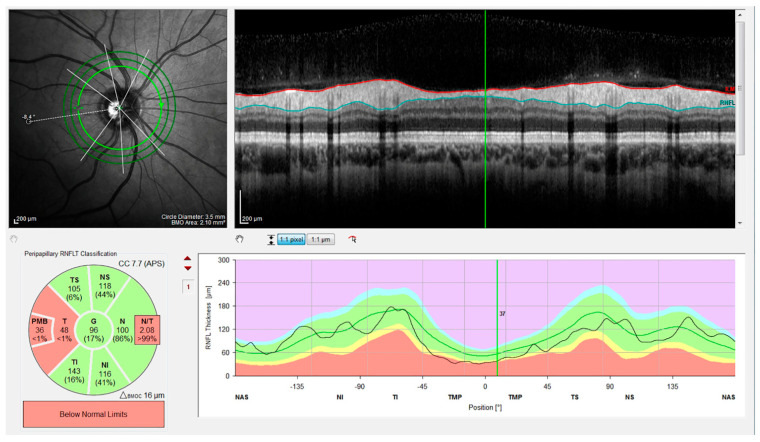
OCT scan of the optic nerve in a patient affected by MS.

In addition, measurements of the peripapillary RNFL thickness (pRNFLt) were performed in all subjects, using the Nsite Analytics™ module of the Spectralis OCT device (Figure 2). pRNFLt was measured around the disk with 16 averaged, consecutive circular B-scans (diameter of 3.5 mm, 768 A-scans), and represented the mean distance between the ILM and the posterior boundary of the RNFL, along a 6° radius circle scan centered on the optic nerve head (ONH) [2].

The Nsite Analytics™ module for Spectralis (version 6.0c, Heyex, Heidelberg Engineering, Heidelberg, Germany) provides a unique analysis of the pRNFLt. Based on the Nsite normative database, a classification color scheme indicates not only axonal loss, but also edematous changes. The software provided a focused analysis of the papillomacular bundle (NITSN-Scan), and a fovea-to-disk alignment (FoDi) to ensure anatomically accurate start/stop of NITSN data of each patient, helping to minimize variability due to patient head orientation. Analysis was based uniquely on high-quality scans, defined as scans with signal quality of >25, without discontinuity or misalignment, poor illumination, involuntary saccades, or blinking artifacts and absence of algorithm segmentation failure on careful visual inspection [2]. No manual correction to the Spectralis automatic segmentation of the different retinal layers was required.

An internal fixation target was used, as this method is reported to have the highest reproducibility.

The thickness of the pRNFL, subdivided into six quadrants, was measured using RNFL-N axonal analytics: temporal (T), temporal–superior (TS), nasal–superior (NS), nasal (N), nasal–inferior (NI), and temporal–inferior (TI). Moreover, the global thickness of the pRNFL (G), the thickness of the papillo-macular bundle (PMB), and the ratio between the thickness of the nasal and temporal quadrant (N/T) were also assessed.

To acquire the posterior pole and pRNFL scans, the patients were asked to fixate on a central target and a nasal target, respectively [8].

Statistical analysis was performed using SPSS version 15.0 (SPSS, Chicago, IL, USA). Each eye was considered a statistical unit. Eyes of RRMS patients previously affected by ON were excluded from the analysis. Comparisons were made between MS and control groups. Histograms and the Kolgomorov–Smirnov test were used to verify Gaussian distributions; mean and standard deviation were applied to parameters responding to Gaussian distribution. Comparisons between groups were performed through one-way analysis of variance (ANOVA), and comparisons between frequencies by Chi-squared tests and Fisher’s exact test in the case of frequencies of <5. Correlations between SD–OCT parameters (PP, the thickness of each retinal layer, and pRNFLt), demographic, and neurological data (EDSS, disease duration) were calculated by linear regression analysis (r Pearson coefficient), in addition to nominal and ordinal multivariate regression analyses. A *p*-value of <0.05 was considered statistically significant.

## 3. Results

### 3.1. Demographic and Clinical Variables

53 RRMS patients and 53 control individuals were included in the study. In total, 97 MS eyes and 106 control eyes were analyzed. Nine eyes from the MS group were excluded due to the previous history of optic neuritis. Demographic and clinical variables of the MS patients and controls are described in Table 1. Groups were matched for age and gender (*p* > 0.05).

### 3.2. OCT Variables

#### 3.2.1. Mean Thickness of the Retinal Posterior Pole by Layers

Overall mean thickness of retinal posterior pole was found not to be different (*p* = 0.3) when comparing MS patients (mean 291.6 ± 12.2 µ) and the control group (293.2 ± 12.7 µ), showing that the total final retinal thickness volume was not globally impaired. However, looking at each layer individually, a complex reorganization of the retinal structure was observed. In MS eyes, we found a decrease in the mean thickness of ppIRLs compared to the control group, which was statistically significant for ppGCL (MS = 31.4 ± 2.6 µ, control = 32.7 ± 1.9 µ, *p* < 0.001), and showed a trend toward significance for ppRNFL (MS = 41.6 ± 5.2 µ, control = 42.8 ± 4.5 µ, *p* = 0.072) and ppIPL (MS eyes 27.2 ± 2 µ, control 26.7 ± 1.6 µ, *p* = 0.074). Conversely, we found a thickening of ppORL. In particular, the mean ppRPE was found to be significantly increased in the MS group (MS = 13.5 ± 1 µ, control 13.1 ± 1.1 µ, *p* = 0.014), as well as ppPHOTO (MS = 77.6 ± 2.2 µ, control 76.8 ± 2.1 µ, *p* < 0.001). No difference (*p* > 0.5) in mean thickness was detected in the remaining layers (ppIPL, ppINL, ppOPL, ppONL) (Table 2).

#### 3.2.2. Mean Thickness of the Retinal Posterior Pole by Layers and Regions

**Figure 3:**
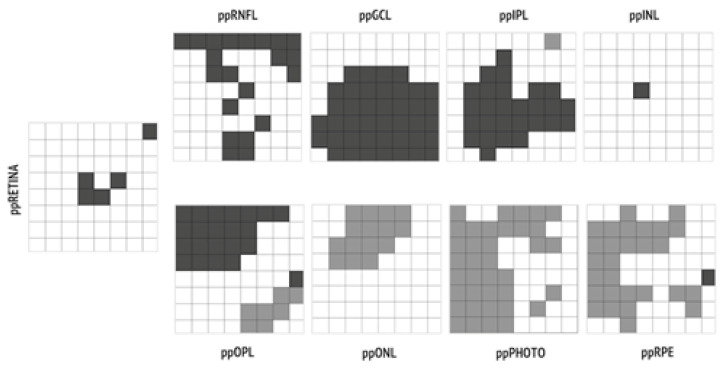
Regions of interests characterized by statistically significant (*p* < 0.05) thinning in MS eyes versus control eyes are represented in dark grey; regions characterized by statistically significant (*p* < 0.05) thickening are represented in light grey.

In comparing each of the 64 volumetric units of each posterior pole layer of the MS and control groups, we found that regions within the same layer were not homogeneously affected by pathological rearrangement, as shown in Figure 3.

#### 3.2.3. Mean Thickness of the Optic Disk by Quadrants

PMB thickness (MS = 49.8 ± 8.9 µ, control = 54.3 ± 6.9 µ, *p* = 0.0001) and T thickness (MS 65.7 ± 12.2 µ, control 71.1 ± 9.8 µ, *p* = 0.001) were significantly decreased in MS patients, compared to the control group. Furthermore, the MS group showed a significantly increased N/T ratio (MS = 1.3 ± 0.5, control 1.19± 0.24, *p* = 0.017, *p* = 0.017) (Table 3).

### 3.3. Correlations between OCT Parameters and Clinical Parameters

Considering the retinal posterior pole, a significant inverse correlation between disease duration and retina (r = −0.390, *p* < 0.001), ppRNFL (r = −0.423, *p* < 0.001), ppGCL (r = −0.365, *p* < 0.001) was found. Considering the ONH, significant inverse correlations between disease duration and peripapillary macular bundle (PMB) (r = −0.501, *p* < 0.001), temporal–superior (TS) (r = −0.231, *p* = 0.02), temporal (T) (r = −0.463, *p* < 0.001), temporal–inferior (TI) (r = −0.317, *p* = 0.002), and global (G) (r = −0.316, *p* = 0.02) regions were found. Interestingly, EDSS was negatively correlated with ppRNFL (r = −0.388, *p* < 0.001), and positively correlated with ppINL (r = 0.208, *p* = 0.041). At ONH level, EDSS was inversely correlated to PMB (r = −0.267, *p* = 0.008), TS (r = −0.280, *p* = 0.005), T (r = −0.345, *p* = 0.001), TI (r = −0.245, *p* = 0.016), and G (r = −0.264, *p* = 0.009) (Table 4).

## 4. Discussion

Visual impairment is a common feature in MS, mainly subtended by inflammatory-mediated axonal degeneration and neuronal loss. The optic nerve is not the only visual region involved; in fact, the retina also shows variable degrees of damage [1]. SD-OCT examination is a fast, non-invasive, and reproducible method that allows for obtaining an optical biopsy of the retina, whose role in detecting neuroaxonal degeneration in MS is increasingly acknowledged. Nevertheless, previous studies using OCT have focused only on the assessment of pRNFL and the macular region, mainly due to technical limitations. In our study, the OCT Spectralis automated segmentation software has been applied to explore the structural damage of the entire retinal posterior pole in MS patients.

The results of our study highlighted that, in MS, neurodegeneration is not limited to the optic nerve head and the peripapillary areas, but involves a larger retinal area. According to our data, the structural remodeling does not equally affect all the retinal layers and all the sub-regions of the posterior pole. There is, in fact, predominant neurodegeneration in the ppIRLs, while the ppORLs appear thickened.

Remarkably, ppGCL thinning predominantly accounts for IRL reduction. This finding is consistent with previous evidence showing a thinning of the ganglion cell–inner plexiform layer complex at the macular level [1,9,10]. Nevertheless, we were able to demonstrate that GCL damage spreads in the entire retinal posterior pole. We also found a slight and not statistically significant reduction of ppIPL. GCL is mainly composed of retinal ganglion cells (RGCs), which demand a high energy supply, and whose survival and function depend upon adenosine triphosphate (ATP), differently from photoreceptors [11]. Therefore, RGCs may be distinctively susceptible to MS-related neurodegeneration, due to the deficiency of the mitochondrial respiratory chain complex, limiting the capacity of generating ATP, which is a well-known phenomenon in the pathophysiology of MS [12]. In addition to GCL, previous studies have shown that pRNFL is usually damaged in MS, and pRNFL is now considered one of the most sensitive biomarkers of retinal damage in MS patients with and without ON [13,14]. In our sample, ppRNFL was found only modestly reduced in MS. Nevertheless, it is known that RNFL thinning is increasingly prominent in more advanced stages of the disease, although already visible in the clinically isolated syndrome [15,16]. In this cohort of MS patients, disease duration was relatively short; moreover it was found to be inversely correlated with ppRNFL and ppGCL thickness. Based on these observations, we expect that an overt ppRNFL change will appear in a longitudinal follow-up. A significant inverse correlation between ppRNFL and EDSS has been shown by our results, confirming the validity of RNFL thickness as a biomarker of disability.

In contrast with the thinning of ppIRL, we found an increased thickness of the ppORL, namely of ppRPE and ppPHOTO.

While INLs are made up of cell bodies and axons of RGCs, which are known to be vulnerable to hypoxic damage, the ORLs seemingly have the capacity to react to this damage in an attempt to maintain the homeostasis and structural integrity of the retina. This phenomenon occurs physiologically in the case of mechanical, inflammatory, and metabolic insults, and is predominantly sustained by Müller cells. In MS patients, it is possible that Müller cells try to counterbalance the neurodegenerative process, depositing new organic matrix—consisting mainly of fibrillar proteins—in the intercellular space. These modifications promote retinal tissue repair, and protect the retina from the extension of damage [17,18,19].

Building on a technique which is commonly used for the evaluation of retinal involvement in glaucoma patients (posterior pole analysis), we explored the individual behavior of each retinal layer in MS patients compared to a control. We observed a non-homogeneous neurodegeneration within the same layer, particularly ppRNFL, ppGCL, and ppIPL, that distinctly mark MS from other diseases (i.e., ADOA), in which neurodegeneration is homogeneously distributed [3]. Trophic interdependency of contiguous regions may explain our findings, although we cannot rule out that differences in cellular or vascular components across retinal areas may also play a role [20]. Moreover, the impact of lesion distribution in the central optic pathways was not explored in this study, limiting the interpretation of our results.

Looking at more classical parameters of retinal damage in MS, we found that PMB and the temporal region of the optic nerve were selectively affected by neurodegeneration, with a consequent inversion of the N/T ratio. These data, which are consistent with those previously reported [1], corroborate the accuracy of the OCT acquisition in this study.

In summary, in this work, we showed that neurodegenerative phenomena in MS are not limited to the optic disk and the PMB, expanding far beyond it into the entire retinal posterior pole, in particular into the ppINLs; ppONLs, conversely, appear thickened, presumably in response to ppINL collapse. Heterogeneous involvement of individual retinal regions differentiates MS from other retinal diseases, whose reasons need to be elucidated by future studies. Posterior pole analysis may be a useful method for estimating retinal reorganization of the eye in MS patients in the early stages of the disease.

## 5. Conclusions

To our knowledge, this is the first paper showing that the degenerative process affecting the optic nerve and macular area in patients diagnosed with relapsing remitting multiple sclerosis also extends to the entire posterior pole.

This paper suggests that multiple sclerosis can produce neurodegenerative phenomena that are not limited to the optic disk and the PMB, but extend far beyond it into the entire retinal posterior pole, in particular into the ppINLs. Conversely, ppONLs appear thickened, presumably in response to ppINL collapse. This heterogeneous involvement of individual retinal regions differentiates multiple sclerosis from other retinal diseases. Moreover, our results suggest that posterior pole analysis may be a useful method for estimating retinal reorganization of the eye in multiple sclerosis patients in the early stages of the disease.

## Figures and Tables

**Table 1:** Demographic and clinical data of relapsing remitting MS patients and the control group.

	RRMS	CONTROLS
Eyes (*n*)	97	106
Age (years), mean, SD	37.4 ± 10.1	36.11 ± 12.94
Sex (F), *n*, %	73 F (76.2%)	72 F (67.9%)
EDSS	1.9 ± 1.3	-
Disease duration (months)	43.2 ± 66.7	-

**Table 2:** Posterior pole retinal layers thickness values and statistical differences between patients with MS and the control group.

Group	Thickness (µn)	Retina	RNFL	GCL	IPL	INL	OPL	ONL	PHOTO	RPE
MS	Mean	291.6	41.6	31.4	27.2	30.8	27.1	56.3	77.6	13.5
	SD	12.2	5.2	2.6	2	1.6	2	6.3	2.2	1
CTRL	Mean	293.2	42.8	32.7	26.7	30.7	27.3	55.5	76.8	13.1
	SD	12.7	4.5	1.9	1.6	1.7	2.1	5.4	2.1	1.1
*p*		0.3	0.072	0.001	0.074	0.7	0.7	0.3	0.001	0.014

Retina: total retinal thickness; RNFL: retinal nerve fiber layer; GCL: ganglion cell layer; IPL: inner plexiform layer; INL: inner nuclear layers; OPL: outer plexiform layer; ONL: outer nuclear layer; RPE: retinal pigment epithelium; IRL: inner retinal layers; ORL: outer retinal layers; SD: standard deviation; *p*: significant difference (*p* < 0.05) in ANOVA test between control and MS groups for each population.

**Table 3:** Peripapillary RNFL thickness values provided by RNFL-N axonal analytics and statistical differences in autosomal dominant optic atrophy (ADOA) and control subjects.

Group	Thickness (µn)	PMB	TI	T	TS	NI	N	NS	G	N/T
MS	Mean	49.8	146.6	65.7	125	112.6	83.6	113.4	97.2	1.3
	SD	8.9	21.5	12.2	22.6	23.3	21.8	24.4	12.6	0.5
CTRL	Mean	54.3	150.4	71.1	127.7	110.1	82.9	116.6	99.1	1.19
	SD	6.9	17.2	9.8	17.3	23.3	11.1	19.8	8.0	0.24
*p*		0.0001	0.162	0.001	0.343	0.439	0.783	0.311	0.193	0.017

PMB: papillomacular bundle; TI: temporal–inferior; T: temporal; TS: temporal–superior; NI: nasal–inferior; N: nasal; NS: nasal–superior; G: global; N/T: nasal-to-temporal ratio; SD: standard deviation; *p*: significant difference (*p* < 0.05) in ANOVA tests between control and MS groups for each population.

**Table 4:** Correlations (R) between OCT parameters and clinical parameters.

		Retina	RNFL	GCL	PMB	TI	T	TS	G
Disease duration	R	−0.390	−0.423	−0.365	−0.501	−0.317	−0.463	−0.231	−0.316
	*p*	0.001	0.001	0.001	0.001	0.002	0.001	0.020	0.020
EDSS	R		−0.388		−0.267	−0.245	0.345	−0.280	−0.264
	*p*		0.001		0.008	0.016	0.001	0.005	0.009

## Data Availability

Data will be available upon reasonable request to the corresponding author.

## References

[1] Petzold A., Balcer L.J., Calabresi P.A., Costello F., Frohman T.C., Frohman E.M., Martinez-Lapiscina E.H., Green A.J., Kardon R., Outteryck O. (2017). Retinal layer segmentation in multiple sclerosis: A systematic review and meta-analysis. Lancet Neurol..

[2] Cesareo M., Ciuffoletti E., Martucci A., Balducci C., Cusumano A., Ricci F., Sorge R. (2016). Automatic Segmentation of Posterior Pole Retinal Layers in Patients with Early Stage Glaucoma Using Spectral Domain Optical Coherence Tomography. J. Clin. Exp. Ophthalmol..

[3] Cesareo M., Ciuffoletti E., Martucci A., Sebastiani J., Sorge R.P., Lamantea E., Garavaglia B., Ricci F., Cusumano A., Nucci C. (2017). Assessment of the retinal posterior pole in dominant optic atrophy by spectral-domain optical coherence tomography and microperimetry. PLoS ONE.

[4] Martucci A., Toschi N., Cesareo M., Giannini C., Pocobelli G., Garaci F., Mancino R., Nucci C. (2018). Spectral Domain Optical Coherence Tomography Assessment of Macular and Optic Nerve Alterations in Patients with Glaucoma and Correlation with Visual Field Index. J. Ophthalmol..

[5] Chorostecki J., Seraji-Bozorgzad N., Shah A., Bao F., Bao G., George E., Gorden V., Caon C., Frohman E., Bhatti M.T. (2015). Characterization of retinal architecture in Parkinson’s disease. J. Neurol. Sci..

[6] Murphy O.C., Kalaitzidis G., Vasileiou E., Filippatou A.G., Lambe J., Ehrhardt H., Pellegrini N., Sotirchos E.S., Luciano N.J., Liu Y. (2020). Optical Coherence Tomography and Optical Coherence Tomography Angiography Findings After Optic Neuritis in Multiple Sclerosis. Front Neurol..

[7] Polman C.H., Reingold S.C., Banwell B., Clanet M., Cohen J.A., Filippi M., Fujihara K., Havrdova E., Hutchinson M., Kappos L. (2011). Diagnostic criteria for multiple sclerosis: 2010 revisions to the McDonald criteria. Ann. Neurol..

[8] Schuman J.S., Pedut-Kloizman T., Hertzmark E., Hee M.R., Wilkins J.R., Coker J.G., Puliafito C.A., Fujimoto J.G., Swanson E.A. (1996). Reproducibility of nerve fiber layer thickness measurements using optical coherence tomography. Ophthalmology..

[9] Garcia-Martin E., Polo V., Larrosa J.M., Marques M.L., Herrero R., Martin J., Ara J.R., Fernandez J., Pablo L.E. (2014). Retinal layer segmentation in patients with multiple sclerosis using spectral domain optical coherence tomography. Ophthalmology.

[10] Britze J., Pihl-Jensen G., Frederiksen J.L. (2017). Retinal ganglion cell analysis in multiple sclerosis and optic neuritis: A systematic review and meta-analysis. J. Neurol..

[11] Yu D.Y., Cringle S.J., Balaratnasingam C., Morgan W.H., Yu P.K., Su E.N. (2013). Retinal ganglion cells: Energetics, compartmentation, axonal transport, cytoskeletons and vulnerability. Prog. Retin. Eye Res..

[12] Campbell G., Licht-Mayer S., Mahad D. (2019). Targeting mitochondria to protect axons in progressive MS. Neurosci. Lett..

[13] Parisi V., Manni G., Spadaro M., Colacino G., Restuccia R., Marchi S., Pierelli F. (1999). Correlation between morphological and functional retinal impairment in multiple sclerosis patients. Investig. Ophthalmol. Vis. Sci..

[14] Saidha S., Al-Louzi O., Ratchford J.N., Bhargava P., Oh J., Newsome S.D., Prince J.L., Pham D., Roy S., van Zijl P. (2015). Optical coherence tomography reflects brain atrophy in multiple sclerosis: A four-year study. Ann. Neurol..

[15] Narayanan D., Cheng H., Bonem K.N., Saenz R., Tang R.A., Frishman L.J. (2014). Tracking changes over time in retinal nerve fiber layer and ganglion cell-inner plexiform layer thickness in multiple sclerosis. Mult. Scler..

[16] Gelfand J.M., Goodin D.S., Boscardin W.J., Nolan R., Cuneo A., Green A.J. (2012). Retinal axonal loss begins early in the course of multiple sclerosis and is similar between progressive phenotypes. PLoS ONE.

[17] Manogaran P., Samardzija M., Schad A.N., Wicki C.A., Walker-Egger C., Rudin M., Grimm C., Schippling S. (2019). Retinal pathology in experimental optic neuritis is characterized by retrograde degeneration and gliosis. Acta Neuropathol. Commun..

[18] Langhe R., Pearson R.A. (2020). Rebuilding the Retina: Prospects for Müller Glial-mediated Self-repair. Curr. Eye Res..

[19] Ziccardi L., Barbano L., Boffa L., Albanese M., Grzybowski A., Centonze D., Parisi V. (2020). Morphological Outer Retina Findings in Multiple Sclerosis Patients with or Without Optic Neuritis. Front. Neurol..

[20] Nuschke A.C., Farrell S.R., Levesque J.M., Chauhan B.C. (2015). Assessment of retinal ganglion cell damage in glaucomatous optic neuropathy: Axon transport, injury and soma loss. Exp. Eye Res..

